# The Role of Desflurane in the Functional Outcomes Among Spinal Cord Injury Patients Undergoing Upper Extremity Nerve Transfer Procedures

**DOI:** 10.7759/cureus.79447

**Published:** 2025-02-22

**Authors:** Muhammad I Kaleem, Arbi B Abdallah, Saad Javeed, Sahasraara Hemanth, Daniel M Hafez, Jacob K Greenberg, Wilson Z Ray, Umeshkumar Athiraman

**Affiliations:** 1 Neurological Surgery, Washington University School of Medicine, St. Louis, USA; 2 Anesthesiology, Washington University School of Medicine, St. Louis, USA

**Keywords:** functional outcomes, neuroprotection, peripheral nerve transfers, spinal cord injury, volatile anesthetics

## Abstract

Background and objective

While several experimental studies have demonstrated the neuroprotective role of volatile anesthetics after spinal cord injury (SCI), the impact of volatile anesthetics on improving neurologic outcomes in spinal cord-injured patients is not known. Hence, this study aimed to examine the impact of volatile anesthetics on functional outcomes of chronic cervical SCI patients undergoing peripheral nerve transfer procedures.

Methods

We conducted a retrospective analysis involving adult patients with cervical SCI and upper extremity paralysis undergoing nerve transfer procedures between September 1, 2015, and January 31, 2019. The principal outcome measured was the motor strength of the reinnervated muscle targets assessed per the Medical Research Council (MRC) scale graded from 0 to 5. Secondary outcomes included Disabilities of Arm, Shoulder, and Hand (DASH); Sollerman Hand Function Test (SHFT); and Michigan Hand Questionnaire (MHQ) scores. Univariate analysis and logistic regression were performed to examine the association between the volatile anesthetics (sevoflurane, desflurane) used during the nerve transfer surgery with the improvement in muscle strength examined up to 48 months postoperatively.

Results

A total of 13 tetraplegic patients with a mean age of 39.2 ± 15.9 years were included in this study. We found that the desflurane group (n=22 muscles) had greater median motor strength than the sevoflurane group (n=60 muscles) towards the final follow-up when the desflurane group had median motor strength of 3 [interquartile range (IQR): 1-4] and sevoflurane group had a median motor strength of 1 (IQR: 0-2.25); p=0.014. However, there was no statistically significant difference between the two groups in the DASH, SHFT, and MHQ scores. Logistic regression analysis showed that type of nerve transfer and preop AIS (ASIA Impairment Scale) grade were significantly associated with higher odds of improvement of motor power by at least 2 grades.

Conclusions

Our preliminary data show an association between desflurane use and improved motor strength in SCI patients undergoing peripheral nerve transfers. The data suggest that volatile anesthetic conditioning-induced protection observed in preclinical studies may also exist in SCI patients. This will need to be validated in a larger sample size. Examining the therapeutic window, and identifying the molecular mechanisms underlying volatile anesthetic conditioning-induced protection are warranted to aid future translational studies.

## Introduction

Spinal cord injury (SCI) is a debilitating condition that more commonly affects young and healthy individuals, leading to physical and psychosocial impairments and ultimately resulting in a significant loss of quality years of life [1]. The incidence of traumatic SCI is approximately 54 cases per one million, with 18,000 new cases reported annually in the United States [2]. Approximately 302,000 patients are living with some degree of tetraplegia in the United States [2]. It is important to note that less than 1% of patients with traumatic SCI are discharged home with a full neurological recovery [2]. Currently, tendon and nerve transfers are the surgical treatment options available to improve motor recovery and functional outcomes in these patients [3]. Despite significant advancements in the field, complete motor recovery and the functional outcomes after SCI remain poor. Hence, alternate therapeutic strategies that could promote reinnervation to improve functional independence and quality of life in SCI patients are critically needed.

Conditioning is a phenomenon that engages powerful pleiotropic endogenous protective cascades, that have previously been demonstrated to provide protection in several organ systems [4,5]. In particular, numerous preclinical studies have demonstrated the neuroprotective potential of volatile anesthetics in several neurological disorders such as stroke, subarachnoid hemorrhage, traumatic brain injury, and others [6,7,8,9,10,11]. Additionally, several preclinical studies have shown that conditioning with commonly used volatile anesthetics such as isoflurane and sevoflurane provided significant neuroprotection after ischemic SCI [12,13,14,15,16,17,18,19,20]. However, the effects of these volatile anesthetics on SCI patients are not known yet. In light of this, our current study aims to examine the effect of volatile anesthetics (sevoflurane vs. desflurane) on the functional outcomes of tetraplegic patients undergoing peripheral nerve transfer procedures.

## Materials and methods

Study design and participants

This study was a retrospective analysis of prospectively collected data from our previously reported clinical trial of 22 patients with traumatic cervical SCI who underwent peripheral nerve transfers to improve upper extremity function [3]. Inclusion criteria for nerve transfer procedures were as follows: (1) adult patients (18 years or older), (2) an Informed Consent Document (ICD) signed by the patient, (3) cervical SCI resulting in arm and hand functional impairment, with at least preserved spinal accessory nerve, (4) patients with a stable American Spinal Injury Association (ASIA) grade of A, B, or C showing minimal to no evidence of upper extremity functional improvement in the motor examination after at least six months of nonoperative therapy post-injury, (5) International Classification of Surgery of the Hand in Tetraplegia (ICSHT) category 0-4 (not applicable for patients with a diagnosis of central cord syndrome), (6) willing and able to comply with the study protocol, and (7) <60 months from SCI at the time of surgery.

Patients with the following criteria were excluded from the study: (1) active infection at the operative site or systemic infection, (2) any return or ongoing clinical recovery of distal motor function within SIX months after injury, (3) mentally compromised and lacking the capacity for treatment decision making, (4) undergoing long-term steroid therapy, (5) significant joint contractures and/or limitations in passive range of motion in the arm or hand, (6) active malignancy, (7) any systemic disease that would affect the patient's welfare or the research study, (8) pregnant patient, (9) immunologically suppressed or immunocompromised, (10) previous tendon transfers to restore upper extremity function, (11) affective disorder that would make outcome assessment and study participation difficult, and (12) history of brachial plexus injury or systemic neuropathic process.

Written informed consent was obtained from all study participants for prospective data collection. Ethics approval was obtained from the institutional review board at Washington University in St. Louis. Given the aim of this study i.e., to investigate the association between the choice of volatile anesthetics utilized during the nerve transfer procedure (desflurane or sevoflurane) and muscle strength outcomes, only the 13 patients who underwent a nerve transfer procedure with the same anesthetic for all number of surgeries, i.e., either desflurane or sevoflurane, were included. Patients who received both sevoflurane and desflurane, in any order, were excluded. Also, patients who received combined anesthetic (desflurane, or sevoflurane with propofol) or when nitrous oxide (N_2_0) was utilized were excluded from the analysis to avoid the confounding effects of the impact of combined anesthetics on the measured outcomes. The rationale for this is that nerve regeneration following nerve transfers is a long process and nerve regeneration may still be ongoing when a second anesthetic is used, potentially impacting the outcomes of the prior nerve transfer performed with a different anesthetic.

Interventions

All patients underwent single, double, or triple nerve transfers to restore upper extremity function in one or both limbs. Nerve transfers were chosen based on the (1) level of injury, (2) residual motor function per the ICSHT grouping, and (3) electrodiagnostic patterns of motor neuron injury. Donor nerves were selected if they had clinically functional motor strength, defined as a Medical Research Council (MRC) grade of 4-5. Nerve transfer pairings to restore target functions included (1) posterior deltoid motor branch of the axillary nerve to triceps branch of the radial nerve to reanimate elbow extension, (2) supinator branch of the radial nerve to posterior interosseus nerve (PIN) to reanimate hand opening and finger extension, (3) brachialis branch of the musculocutaneous nerve to anterior interosseus nerve (AIN)/flexor digitorum superficialis (FDS) fascicles of the median nerve to reanimate pinch and finger flexion, and (4) for patients with high cervical SCI (C4 level and above, ICSHT group 0), use of the spinal accessory nerve (SAN) to triceps or AIN. The surgical procedure and occupational hand therapy protocols are described in our previous report [3].

Outcomes

Clinical outcomes were assessed preoperatively and at two, six, 12, 18, 24, 36, and 48 months postoperatively. The principal outcome in our current study is the motor strength of each muscle reinnervated per MRC grade. Secondary outcomes included the Disabilities of Arm, Shoulder and Hand (DASH), Sollerman Hand Function Test (SHFT), and Michigan Hand Outcome Questionnaire (MHQ) scores for assessment of function and patient-reported outcomes. DASH is a patient-reported measure consisting of 30 items relating to the ability to perform daily activities and symptoms experienced, each scored from 1 to 5. The total score is then extrapolated on a scale from 0 to 100, with higher scores indicating more disability. SHFT measures hand function as related to daily activities with 20 tasks rated from 0 to 4 depending on the time taken for a given task, to a maximum of 80. Higher SHFT scores signify increased function. MHQ is a patient-reported questionnaire that evaluates the function of each hand in daily living. Raw scores from six individual components (pain component reversed) are normalized to get a total score for each side on a range of 0-100, with higher scores indicating better function.

Statistical analysis

Missing data were handled with the (last observation carried forward" approach. Limited by the sample size and to account for potential variations in reinnervation outcomes across different muscle groups, we assessed each individual muscle reinnervated separately. Categorical variables are presented as numbers (percentages), and continuous data are reported as median [interquartile range (IQR)] or mean [standard deviation (SD)] as appropriate. Median values of muscle strength at each time point, from baseline to final follow-up, and DASH, SHFT, and MHQ scores for the two groups of patients (desflurane and sevoflurane) were compared using the Wilcoxon rank-sum test. The threshold for significance was set at a two-sided α<0.05. Patients with preoperative data not available were excluded from this analysis. The latest follow-up observation was carried forward for the final follow-up (48 months). Binary logistic regression was also applied to examine the association between improvement of muscle strength of at least 2 grades on the MRC scale and type of anesthetic choice (sevoflurane or desflurane), neurological level of cervical SCI (high or low), preop AIS grade (A or B/C/D) and laterality (left or right limb). All data analyses were performed using R, version 4.2.1 (R Foundation for Statistical Computing).

## Results

Demographic data

Thirteen patients with tetraplegia with a median age of 39.2 ± 15.9 years (12 male and one female) were included in this retrospective analysis. Demographic characteristics are presented in Table 1. There were five patients in the desflurane group and eight in the sevoflurane group. The mean time to surgery was 24.2 ± 16.1 months for desflurane and 26.1 ±16.4 months for sevoflurane. The two groups did not differ in terms of age at the time of surgery, time to surgery, sex, race, neurological level of injury, and preop AIS grade. However, their distribution did differ in ICSHT groups. Twenty-eight nerve transfers were performed in 19 procedures. Among these, 14 (50%) were brachialis motor branch to AIN, nine (32%) were supinator motor branch to PIN, two (7%) were SAN to triceps motor branch, and one (3.6%) each was posterior axillary nerve (deltoid motor branch) to triceps motor branch SAN to AIN and flexor carpi radialis/flexor digitorum superficialis motor fascicle to biceps motor branch (Table 2). Twenty-two muscles (27%) were reinnervated in the desflurane group and 60 (73%) in the sevoflurane group (Table 3).

**Table 1:** Patient characteristics by type of anesthetic used ^*^Two-sided t-test for continuous variables, Fisher’s exact test for categorical variables AIS: the American Spinal Injury Association Impairment Scale; ICSHT: International Classification of Surgery of the Hand in Tetraplegia; SD: standard deviation

Variables		Desflurane	Sevoflurane	Total	P-value^*^
	Total, n (%)	5 (38.5)	8 (61.5)	13	
Age at surgery (years)	Mean ± SD	37.1 ± 11.1	40.5 ± 19.0	39.2 ± 15.9	0.69
Time to surgery from injury (months)	Mean ± SD	24.2 ± 16.1	26.1 ± 16.4	25.4 ± 15.6	0.84
Sex	Female	1 (20.0)	0 (0.0)	1 (7.7)	0.38
	Male	4 (80.0)	8 (100.0)	12 (92.3)	
Race	Black or African American	0 (0.0)	0 (0.0)	0 (0.0)	1
	White or Caucasian	5 (100).0	8 (100.0)	13 (100.0)	
Neurological level of injury	High	3 (60.0)	4 (50.0)	7 (53.8)	1
	Low	2 (40.0)	4 (50.0)	6 (46.2)	
Preop AIS grade	A (complete)	1 (20.0)	5 (62.5)	6 (46.2)	0.27
	B/C/D (incomplete)	4 (80.0)	3 (37.5)	7 (46.2)	
ICSHT group	0	2 (40.0)	0 (0.0)	2 (16.7)	0.05
	1	0 (0.0)	4 (57.1)	4 (33.3)	
	3	1 (20.0)	3 (42.9)	4 (33.3)	
	4	2 (40.0)	0 (0.0)	2 (16.7)	

**Table 2:** Nerve transfers performed in each anesthetic group AIN: anterior interosseus nerve; FCR: flexor carpi radialis; FDS: flexor digitorum superficialis; PIN: posterior interosseus nerve; SAN: spinal accessory nerve

Nerve transfer	Desflurane, n (%)	Sevoflurane, n (%)	Total, n (%)
Brachialis to AIN	3 (37.5)	11 (55.0)	14 (50.0)
Supinator to PIN	2 (25.0)	7 (35.0)	9 (32.1)
Axillary to triceps	0 (0.0)	1 (5.0)	1 (3.6)
SAN to triceps	2 (25.0)	0 (0.0)	2 (7.1)
SAN to AIN	1 (12.5)	0 (0.0)	1 (3.6)
FDS/FCR to biceps	0 (0.0)	1 (5.0)	1 (3.6)
Total	8 (28.6)	20 (71.4)	28 (100)

**Table 3:** Number of muscles reinnervated in each anesthetic group EDC: extensor digitorum communis; EPB: extensor policis brevis; FCR: flexor carpi radialis; FDP: flexor digitorum profundus; FDS: flexor digitorum superficialis; FPL: flexor policis longus

Muscle	Type of Anesthesia		
	Desflurane, n (%)	Sevoflurane, n (%)	Total, n (%)
EDC	2 (9.1)	7 (11.7)	9 (11.0)
EPB	2 (9.1)	7 (11.7)	9 (11.0)
FCR	4 (18.2)	11 (18.3)	15 (18.3)
FDP	4 (18.2)	11 (18.3)	15 (18.3)
FDS	4 (18.2)	11 (18.3)	15 (18.3)
FPL	4 (18.2)	11 (18.3)	15 (18.3)
Triceps	2 (9.1)	1 (1.7)	3 (3.7)
Biceps	0 (0)	1 (1.7)	1 (1.2)
Total	22 (26.8)	60 (73.2)	82 (100)

Effect of anesthesia type on motor strength outcomes

**Figure 1:**
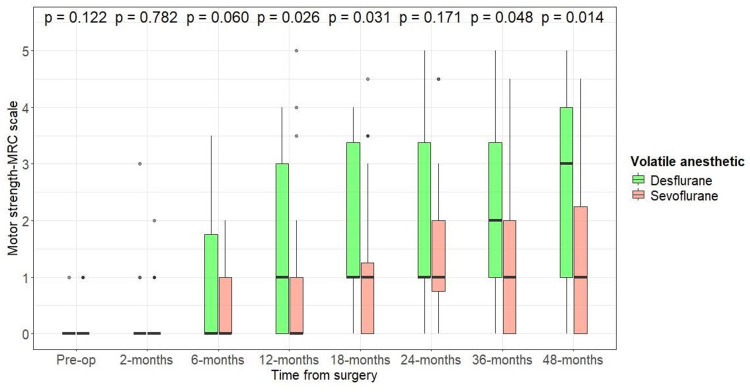
Effect of anesthetic used (desflurane vs. sevoflurane) during nerve transfer procedure on motor strength of reinnervated muscle target Measured per the MRC scale. Desflurane (n=22); sevoflurane (N=60). Wilcoxon rank-sum test, alpha level of significance: <0.05 MRC: Medical Research Council

We used the Wilcoxon rank-sum test to compare the motor power of reinnervated muscles on the MRC scale between patients who received a nerve transfer under desflurane (n=22 muscle targets) or sevoflurane (n=60 muscle targets) (Figure 1). There was no significant difference in median motor strength of reinnervated muscle targets between the two groups before 12 months, when patients operated under desflurane had motor strength of 1 (IQR: 0-3) and patients operated under sevoflurane had motor strength of 0 (IQR: 0-1); p=0.026. At 18 months post-nerve transfer, the desflurane group had a motor strength of 1: (IQR 1-3.4) and the sevoflurane group had a motor strength of 1 (IQR: 0-1.3); p=0.031. A trend of the desflurane group having greater motor strength than the sevoflurane group persisted towards the final follow-up when the desflurane group had a motor strength of 3 (IQR: 1-4) and the sevoflurane group had a motor strength of 1 (IQR: 0-2.25); p=0.014 (Figure 1).

Secondary clinical outcomes

**Figure 2:**
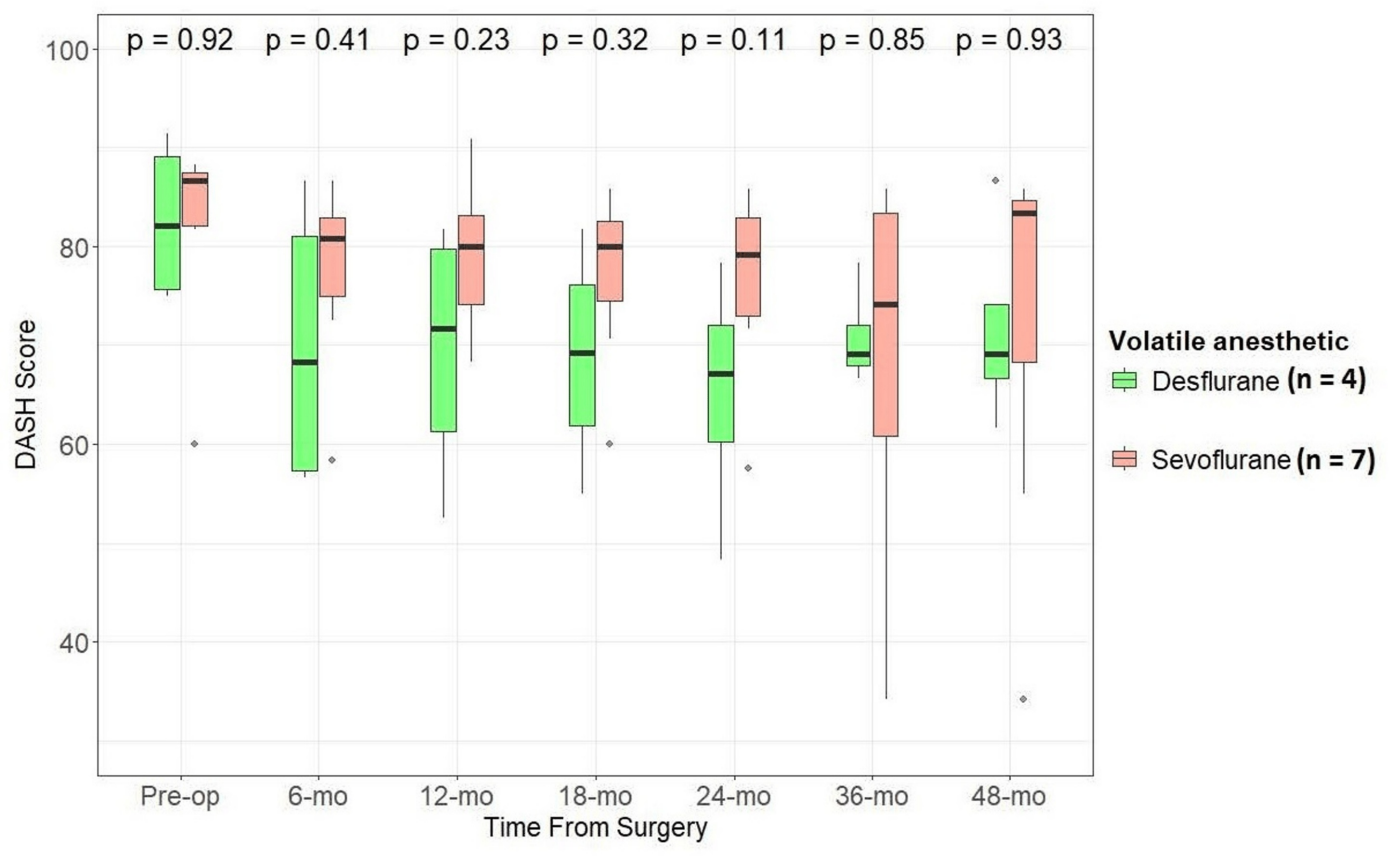
Effect of anesthetic used (desflurane vs. sevoflurane) during nerve transfer procedures on DASH scores through follow-up Wilcoxon rank-sum test, alpha level of significance: <0.05 DASH: Disabilities of Arm, Shoulder, and Hand

**Figure 3:**
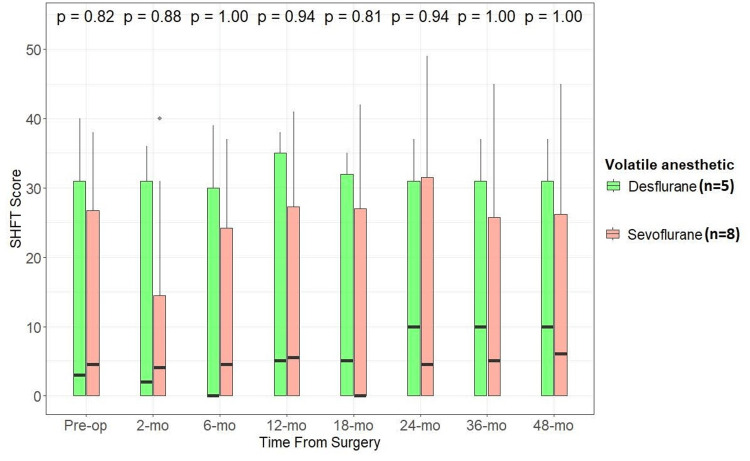
Effect of anesthetic used (desflurane vs. sevoflurane) during nerve transfer procedures on SHFT scores through follow-up Wilcoxon rank-sum test, alpha level of significance: <0.05 SHFT: Sollerman Hand Function Test

**Figure 4:**
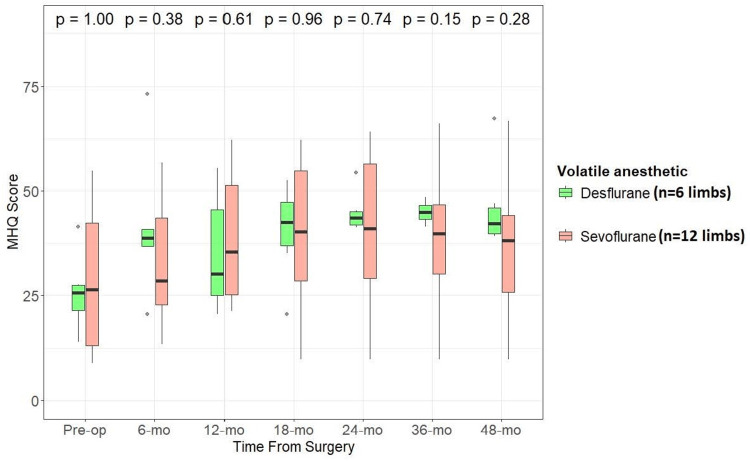
Effect of anesthetic used (desflurane vs. sevoflurane) during nerve transfer procedures on MHQ scores through follow-up Wilcoxon rank-sum test, alpha level of significance: <0.05 MHQ: Michigan Hand Questionnaire

We also examined if there was any difference in functional and patient-reported outcome measures between patients who underwent nerve transfer procedures under sevoflurane compared to desflurane. We compared DASH (lower scores indicate lesser impairment), SHFT (higher scores indicate better function), and MHQ (higher scores indicate better function) scores between the two groups. While median values for all the scores were better for the desflurane group than the sevoflurane group at the latest follow-up, there was no statistically significant difference between the two groups (α -level of significance at 0.05) (Figures 2, 3, 4).

Logistic regression

Observing a difference in motor strength outcome between the desflurane and sevoflurane groups, we investigated other factors that may contribute to the difference in postoperative motor strength. We conducted logistic regression to determine the association between improvement of MRC score by more than 2 grades (no vs. yes) and anesthetic choice (desflurane vs. sevoflurane), neurological level of cervical SCI (low vs. high), preop AIS grade (A vs. B/C/D) and laterality (right vs left limb) at the latest follow-up (Table 4). Odds ratios greater than 1.0 indicate that exposure is associated with a higher chance of motor strength improvement by at least 2 grades on MRC and odds ratios less than 1.0 indicate that exposure is associated with a lower chance of motor strength improvement by at least 2 grades. This model reveals that compared to sevoflurane, odds of improvement of more than 2 grades of motor strength with desflurane were as follows: OR: 2.29 [95% CI: 0.85-6.51], p=0.11; not statistically significant when considered as a single predictor. It was also not significant when accounting for the effect of other factors in the model: OR: 1.57 [95% CI: 0.36-7.04], p=0.55 (Table 4). Factors that were significantly associated with higher odds of improvement of motor power by at least 2 grades included a nerve transfer to PIN (relative to AIN) (OR: 4.66 [95% CI: 1.33-19.02], p=0.02) and AIS grades other than A (relative to A) (OR: 6.22 [95% CI: 1.66-27.06], p=0.01). However, this model could explain only 20% (McFadden Pseudo-R²: 0.21) of variance.

**Table 4:** Single predictor and multivariable logistic regression analysis to explore the association of type of anesthesia, level of cervical SCI, operated side, type of nerve transfer, and preoperative AIS grade with improvement of motor strength by two or more grade Pseudo-R² (McFadden): 0.20, AIS: 104.52 AIN: anterior interosseous nerve; AIS: the American Spinal Injury Association Impairment Scale; ICSHT: International Classification of Surgery of the Hand in Tetraplegia; PIN: posterior interosseous nerve; SCI: spinal cord injury

	Predicted variable: muscle strength on the MRC scale improved by at least 2 grades			
	Single-predictor regression		Multivariable regression	
Predictors	OR (CI)	P-value	OR (CI)	P-value
(Intercept)			0.24 (0.08-0.67)	0.01
Desflurane (vs. sevoflurane)	2.29 (0.85-6.51)	0.11	1.57 (0.36-7.04)	0.55
Low cervical SCI (vs. high cervical SCI)	2.04 (0.85-5.05)	0.11	1.27 (0.33-4.77)	0.72
Right limb (vs. left)	1.65 (0.69-4.01)	0.26	0.68 (0.18-2.31)	0.54
PIN (vs. AIN)	3.90 (1.29-13.48)	0.02	4.66 (1.33-19.02)	0.02
Biceps, triceps (vs. AIN)	4.50 (0.54-93.95)	0.20	3.57 (0.29-91.91)	0.35
Preop AIS grades other than A (vs. grade A)	6.59 (2.59-17.98)	0.00	6.22 (1.66-27.06)	0.01

## Discussion

The main finding in our study is that on univariate analysis, the use of volatile anesthetic desflurane during the upper extremity nerve transfer procedures in SCI patients was associated with improved motor recovery when compared to the patients who received sevoflurane. This finding is notable for a couple of reasons: (1) it may align with the results of previous preclinical studies demonstrating that volatile anesthetic conditioning provides significant neuroprotection after ischemic SCI [12,13,14,15,16,17,18,19,20] and (2) a differential impact may exist within the volatile anesthetics in terms of maximal neuroprotection. Overall, these results suggest that certain volatile anesthetics may have a potential role in improving functional outcomes in SCI patients and the appropriate selection of anesthetics in these patients undergoing general anesthesia may lead to better neurologic outcomes. Findings of multivariable regression suggest that other factors are also important in motor outcomes in nerve transfers. The finding that there are greater odds of improvement in motor strength where the recipient nerve is PIN is consistent with our prior report. The multivariable regression results suggest that a larger sample size is necessary to determine a true association.

The neuroprotective potential of volatile anesthetics has been demonstrated in several preclinical studies on various neurological disorders including SCI [6,7,8,9,10,11,12,13,14,15,16,17,18,19,20]. Specifically, several experimental studies have shown that conditioning with volatile anesthetics such as isoflurane and sevoflurane improved neurologic and histopathologic outcomes after SCI [12,13,14,15,16,17,18,19,20]. Some of the proposed mechanisms for anesthetic conditioning-induced neuroprotection after SCI are (1) activation of mitochondrial adenosine triphosphate-dependent potassium channel, TWIK-related K⁺ channel 1, extracellular signal-regulated kinase; (2) increased expression of nuclear factor kappa B, and inducible nitric oxide synthase; (3) production of free radicals, (4) inhibition of matrix metalloproteinase-9, and cycloxygenase-2 expression, and (5) regulation of the expression of several miRNAs [12,13,14,15,16,17,18,19,20]. Though several preclinical reports exist on this topic, no clinical studies have been reported thus far. To the best of our knowledge, this is the first study to compare the effects of volatile anesthetics on functional outcomes in SCI patients. Interestingly, we noticed an association between desflurane and improved motor function in SCI patients, though the same effect was not observed with sevoflurane.

Evidence shows that the improvement in functional outcomes after nerve transfer procedures is a prolonged process, probably due to the delay in axonal regeneration from the nerve transfer site to the target muscle [3]. Hence, alternate treatments that could either complement the surgical procedures or serve as a stand-alone therapy in promoting nerve regeneration are highly desired and warranted to accelerate functional recovery in SCI patients. Our preliminary data in a sciatic nerve cut repair murine model showed that repetitive exposure to isoflurane (2% for one hour, for three or six consecutive days) improved functional outcomes at 12 weeks post-peripheral nerve injury (PNI) [21]. In addition, the histomorphometric analysis indicated that isoflurane conditioning significantly increased myelin formation, and a nonsignificant increase in axonal count was also noted compared to the control group [21]. These results suggest that certain groups of anesthetics could be explored as an individual or supplemental therapeutic strategy for reinnervation in SCI patients.

The application of volatile anesthetic conditioning to patients with SCI or PNI is highly practical as these anesthetics are relatively safe, used in millions of patients daily, and are already approved by the FDA. However, further experimental studies are required before applying these findings in daily practice. Specifically, addressing the impact of other commonly used volatile and intravenous anesthetics such as desflurane and propofol, and identifying the therapeutic window is essential to find the most ideal anesthetic that could ultimately improve patient outcomes after SCI and PNI. It is also important to note that desflurane is the most potent greenhouse gas among the commonly used volatile anesthetics in clinical use [22]. Hence, the selection of the appropriate volatile anesthetic agents should be based on taking into account the perceived risks and benefits.

It is also known that both spinal cord and peripheral nerve injuries alter the neural circuitry and connectivity, limiting neuroplasticity, and resulting in neurological impairment and delayed recovery [23,24]. In particular, cortical neuroplasticity has been shown to play a critical role in functional recovery after SCI and in peripheral nerve regeneration [23,24]. Therapeutic strategies that could enhance neuroplasticity could potentially improve the functional outcomes after spinal cord and peripheral nerve injuries. Interestingly, our preliminary data examining the impact of isoflurane conditioning in the mouse cortex showed that isoflurane exposure significantly altered the expression of genes related to the neuroplasticity of the brain indicating a potential role of volatile anesthetics in the functional recovery of SCI and PNI [25].

Limitations of the study

Our study has a few limitations, which are summarized below:

(1) Primarily, the results reflect a retrospective analysis of a prospective trial. (2) This was a single-center nonrandomized study with a small sample size. The lack of statistically significant difference in motor power for desflurane in the multivariate regression model is likely due to the smaller sample size. However, it is important to note that when considered as a single predictor, desflurane trends towards higher odds of improvement in motor strength compared to sevoflurane, which points to the need to investigate this phenomenon in a larger sample size. (3) It is also important to note that we are unsure if the functional improvement induced by desflurane in these patients is due to the CNS neuroprotection, or due to the creation of the pro-regenerative environment by the anesthetic. (4) The medication history of the patients was not collected in the study and hence we are unsure if any of the medications used by the patients during the study period could have influenced the current results. (5) Finally, our sample had an under-representation of females as well as black or African American individuals.

## Conclusions

Overall, we conclude that our pilot study examining the impact of volatile anesthetics in SCI patients undergoing nerve transfer procedures shows an association between desflurane use and improved motor function. Further randomized studies with larger sample sizes would help to demonstrate the causation between volatile anesthetics and functional outcomes in SCI patients.
